# Disagreeing Perspectives Enhance Inner-Crowd Wisdom for Difficult (but Not Easy) Questions

**DOI:** 10.1177/09567976251325518

**Published:** 2025-03-24

**Authors:** Philippe P. F. M. Van de Calseyde, Emir Efendić

**Affiliations:** 1 Department of Industrial Engineering and Innovation Sciences, Eindhoven University of Technology; 2 Department of Marketing and Supply-Chain Management, School of Business and Economics, Maastricht University

**Keywords:** perspective taking, estimates, wisdom of the inner crowd, question difficulty, estimate diversity, bracketing

## Abstract

Recently, it has been demonstrated that taking a disagreeing perspective increases the accuracy of inner crowds by enhancing estimation diversity. An insightful commentary reanalyzed the data using maximal random structure models and found no increase in accuracy when taking a disagreeing perspective. These findings present a curious challenge for inner-crowd research and hint at the importance of question variability. Here, we present the results of three preregistered experiments (total *N* = 2,884, with online adult participants from the United States and the United Kingdom) that reconcile these findings by discerning between the ease and difficulty of questions. The results support the notion that taking a disagreeing perspective is beneficial for difficult questions, yet harmful for easier questions. We emphasize that question difficulty is a key factor to consider when evaluating the effectiveness of any intervention designed to improve the accuracy of aggregate estimates through the enhancement of diversity.

When a person guesses the answer to an (estimation) question twice, the average of both guesses is often more accurate than each guess on its own—a phenomenon known as “the wisdom of the inner crowd” (Herzog & Hertwig, 2014; van Dolder & van den Assem, 2018; Vul & Pashler, 2008). In a recent article, Van de Calseyde and Efendić (2022) introduced a strategy that enhanced even further the accuracy of people’s average estimates. Specifically, across multiple experiments, they found that taking a disagreeing perspective when making a second estimate (a) generated more diverse estimates (with lower correlations between first and second estimates), (b) increased bracketing rates (where both estimates land on opposite sides of a question’s true answer), and (c) improved the accuracy of average estimates (vs. simply averaging a person’s first and second guess, or averaging a person’s first guess with a second guess taken from an agreeing perspective).

In an insightful commentary, Fiechter (2024) presented a compelling case that challenges the efficacy of taking a disagreeing perspective to improve the wisdom of inner crowds. Specifically, Fiechter (2024) asserted that the authors failed to incorporate a maximal random-effects structure model when testing whether adopting a disagreeing perspective benefits the accuracy of average estimates. More specifically, although the original authors relied on multilevel models with random intercepts for participants and items (questions), the models should have also included item-level random slopes (because the effects of condition are present across items). Failing to include random slopes when appropriate raises the probability of a Type-I error (Barr et al., 2013; Oberauer, 2022). Consequently, Fiechter (2024) reanalyzed the data using the maximal structure model, opting for Bayesian estimation because of convergence issues in a frequentist setting.

This reanalysis revealed that taking a disagreeing perspective when making a second estimate did not improve the overall accuracy of average estimates. These results pose a curious challenge: Taking a disagreeing perspective is an intervention aimed at boosting the accuracy of inner crowds by deanchoring individuals from their first estimates in order to increase the diversity and bracketing rates between both estimates (Herzog & Hertwig, 2014; Vul & Pashler, 2008). Indeed, the original authors reported that both diversity across estimates and bracketing rates were much higher when making a second guess from a disagreeing perspective. Both of these findings typically ought to have led to an improvement in the accuracy of inner crowds; a rich history across various fields, from collective intelligence to cognitive psychology, has found that greater diversity in estimates and higher bracketing rates typically result in more accurate average estimates (Frey & van de Rijt, 2021; Larrick & Soll, 2006; Vul & Pashler, 2008). Yet Fiechter’s detailed analysis shows that item diversity plays an important role, suggesting that perhaps only some items may have been driving the benefit of perspective taking on the inner crowd.

Compelled by Fiechter’s (2024) reanalysis and the importance of understanding the benefit of perspective taking as a viable technique to boost the wisdom of the inner crowd, we set out to understand what could explain these findings. Specifically, we wondered whether there is no benefit to taking a disagreeing perspective under any circumstances or whether an important moderator related to item variability needs to be identified that is relevant for when perspective taking can be beneficial for the wisdom of the inner crowd.

Although not addressed in the commentary, the original article reported results from an experiment in which taking a disagreeing perspective effectively backfired (i.e., in situations where second estimates were likely to be made in the wrong direction; Van de Calseyde & Efendić, 2022). Combining initial estimates with second estimates that are radically different does not, by definition, result in accurate average estimates, suggesting that there may be cases in which adding diversity between estimates may actually be ineffective or even harmful. In what follows, we aim to demonstrate that differences in question difficulty gives rise to these cases. For example, in the case of relatively easy questions (when first estimates are typically already close to the question’s true answer), adding a second guess that greatly moves away from the first one will only harm the accuracy of the average estimate. In contrast, in the case of relatively difficult questions (when first estimates are relatively far removed from the question’s true answer), adding a second guess that is radically different from the first one will arguably benefit the average estimate. Fiechter (2024) similarly hinted at this possibility by pointing out that for some individual items considered in the reanalysis (i.e., where first estimates were generally far removed from the question’s true answer), taking a disagreeing perspective was, in fact, beneficial.

**Statement of Relevance**

Many decisions rest on people’s ability to make accurate estimates, and a key challenge is to identify strategies that improve their accuracy. One such strategy involves the aggregation of multiple estimates from the same person. Research consistently shows that aggregating a person’s initial estimate with a second estimate that is sufficiently diverse results in an average estimate that is more accurate than each estimate on its own. However, although introducing diversity between estimates often results in more accurate aggregate estimates, it does not necessarily imply that following such a strategy is always advantageous. Indeed, the current analyses reveal that aggregating more diverse estimates is beneficial when people address difficult questions (when first estimates are typically far removed from the question’s true answer), yet harmful when they answer easy questions (when first estimates are typically already close to the question’s answer). The difficulty of a question thus seems to be a key factor to consider when implementing interventions designed to enhance the diversity among estimates.

## Research Transparency Statement

### General disclosures

**Conflicts of interest:** All authors declare no conflicts of interest. **Funding:** This research was not supported by a funding agency. **Artificial intelligence:** ChatGPT was used on some sections of the text to improve grammar and to rephrase certain sections. No other artificial-intelligence-assisted technologies were used in this research or the creation of this article. **Ethics:** Ethical approval was obtained from the ethical review board at Eindhoven University of Technology (Reference No. ERB2020IEIS29).

### Experiment 1a disclosures

**Preregistration:** The hypotheses and methods were preregistered, and the analysis plan was partly preregistered (https://doi.org/10.17605/OSF.IO/A8DEH) on December 7, 2023, prior to data collection, which began on December 7, 2023. The analyses discerning the relative ease versus difficulty of questions were not preregistered. There were two minor deviations from the preregistered plan. First, the preregistered models for the interaction test included a random effect of item difficulty for items, but item difficulty varies for participants, not items. The models in the manuscript correctly specify the random structure. Second, the preregistration specified a frequentist mixed-effects model with *lmer*, whereas a Bayesian mixed model with *brms* was used in the manuscript. Note that the preregistered power analysis was based on a planned recruited sample, but the analyzed sample was different because of exclusions. **Materials:** All study materials are publicly available (https://doi.org/10.17605/OSF.IO/SZRHD). **Data:** All primary data are publicly available (https://doi.org/10.17605/OSF.IO/68E4P). **Analysis scripts:** All analysis scripts are publicly available (https://doi.org/10.17605/OSF.IO/GJBKQ). **Computational reproducibility:** The computational reproducibility of the results has been independently confirmed by the journal’s STAR team.

### Experiment 1b disclosures

**Preregistration:** The hypotheses and methods and analysis plan were preregistered (https://doi.org/10.17605/OSF.IO/4AGXJ) on April 2, 2024, prior to data collection, which began on April 4, 2024. There was one minor deviation from the preregistered plan. The preregistered models for the interaction test included a random effect of item difficulty for items, but item difficulty varies for participants, not items. The models in the manuscript correctly specify the random structure. Note that the preregistered power analysis was based on a planned recruited sample, but the analyzed sample was different because of exclusions. **Materials:** All study materials are publicly available (https://doi.org/10.17605/OSF.IO/SZRHD). **Data:** All primary data are publicly available (https://doi.org/10.17605/OSF.IO/68E4P). **Analysis scripts:** All analysis scripts are publicly available (https://doi.org/10.17605/OSF.IO/GJBKQ). **Computational reproducibility:** The computational reproducibility of the results has been independently confirmed by the journal’s STAR team.

### Experiment 2 disclosures

**Preregistration:** The hypotheses and methods and analysis plan were preregistered (https://doi.org/10.17605/OSF.IO/C7VJF) on April 11, 2024, prior to data collection, which began on April 12, 2024. There was one minor deviation from the preregistered plan. The preregistered models for the interaction test included a random effect of item difficulty for items, but item difficulty varies for participants, not items. The models in the manuscript correctly specify the random structure. **Materials:** All study materials are publicly available (https://doi.org/10.17605/OSF.IO/SZRHD). **Data:** All primary data are publicly available (https://doi.org/10.17605/OSF.IO/68E4P). **Analysis scripts:** All analysis scripts are publicly available (https://doi.org/10.17605/OSF.IO/GJBKQ). **Computational reproducibility:** The computational reproducibility of the results has been independently confirmed by the journal’s STAR team.

## Experiments 1a and 1b

### Method

#### Participants and procedure

Using Prolific, we recruited 1,201 participants for Experiment 1a and 1,200 participants for Experiment 1b, to enable us to detect a small general effect size of *d* = 0.20 with 85% power. Participants were paid a fixed amount for their participation. We limited the participant pool to individuals from the United Kingdom and the United States with English as their first language, and we excluded people from joining the study who had participated in similar experiments we ran on the Prolific platform. Following our preregistration plans, we excluded participants from the analyses who reported that they had looked up any of the answers,^1^ resulting in a final sample of 1,193 participants from the United Kingdom and the United States for Experiment 1a (age: *Mdn* = 42 years, interquartile range, or IQR = 22 years; 56% female) and a final sample of 1,196 participants from the United Kingdom and the United States for Experiment 1b (age: *Mdn* = 40 years, IQR = 22 years; 56% female). The two experiments were identical barring a difference in a preregistered analysis (see section on “Question difficulty” for more details). Each experiment included two between-subjects experimental conditions: the self-perspective condition versus the disagreeing-perspective condition. In both conditions, participants were instructed to answer several estimation questions twice (note that participants were not told at the beginning that they had to make a second estimate). Each question’s answer was a percentage, ranging from 0% to 100% (e.g., “What percentage of the world’s tea production is produced in China?”), and participants were able to indicate their answer using a slider that ranged from 0% to 100%. Ethical approval was obtained from the ethical review board at Eindhoven University of Technology.

In the self-perspective condition, for their second estimate, participants were instructed to make a second guess. Specifically, the instruction was: “We will now ask you to provide a second guess at the answer to each of the questions you were asked in the first session. These answers should not be the same as your previous answers: these should reflect your ‘second guess.’” In the disagreeing-perspective condition, for their second estimate, participants were instructed, “Now picture a friend whose views and opinions are very different from yours. To illustrate, when discussing politics, you often find yourself disagreeing on various issues. How would he or she answer these questions? Please answer these questions again, but now as this friend.” Besides these overall instructions, the instructions for second guesses and disagreeing were also displayed to participants for each question. The experimental instructions were identical to the ones used by Van de Calseyde and Efendić (2022).

A remote but untested possibility is that the original experiments presented by Van de Calseyde and Efendić (2022) did not have sufficient power to detect an effect when models with a maximal random-effects structure are used (Fiechter, 2024). Indeed, the original experimental designs were not powered with a multilevel model framework in mind. Primarily, this relates to the number of items (questions) participants respond to. For example, following Judd et al. (2017), Study 1a in the original article (880 participants, two between-subject conditions, and 10 items) had only 27% power to detect an effect of *d* = 0.30 when analyzed with a maximal structure model. The other studies included between 6 and 12 items. Thus, a substantial increase in the number of items would be necessary to get a more accurate sense of the effect’s stability.

To accommodate such a substantial increase in items, both experiments employed an R(NCC) design (Judd et al., 2017). Specifically, to fit a fully maximal random-effects mixed-effect model, Judd et al. (2017) reported that one needs a substantial number of items (at least 120) to obtain a reasonable degree of power (> 80%) to detect a conventionally small effect.^2^ However, as it is not feasible or desirable for a single participant to respond to 120 questions (twice, in our case), Judd et al. (2017) suggested creating blocks of “replications”: Instead of asking all participants to respond to all questions, a subset of participants responds to a subset of questions. Such a design ensures that a study is properly powered (> 80%) without overburdening participants. We therefore first devised 120 estimation questions. We subsequently created 12 replication blocks, each with 10 unique questions (randomly drawn and paired from the overall set of 120 questions). Thus, after being randomly assigned to one of the experimental conditions (self-perspective condition vs. disagreeing-perspective condition), each participant was randomly assigned to one of 12 replication blocks, with each block including 10 questions that a participant answered twice. (All questions and answers used in the experiment are in an Excel file attached to the preregistration of Experiment 1a.) After responding to the questions, participants were asked to provide their age and gender. In addition, they were presented with a question asking them whether they looked up any of the answers to the questions.

#### Measurements and analysis

To demonstrate the benefit, or lack thereof, of taking a disagreeing perspective, we relied on differential mean-squared error (MSE; Vul & Pashler, 2008). We compared, between conditions (i.e., self-perspective condition vs. disagreeing-perspective condition), the difference in MSE between the first guess and the average of the first and second guess. Larger values indicate greater benefit. Similar to Fiechter (2024), we used a maximal Bayesian model using the *brms* package in R (Bürkner, 2017) to avoid any nonconvergence issues when applying a maximal random structure model.

#### Question difficulty

We operationalized question difficulty as the MSE observed in the first estimates of a question. The higher this error (i.e., with first estimates generally diverging considerably from the question’s actual answer), the more difficult the question. After collecting the data, we ranked all 120 items from easiest to most difficult on the basis of the MSEs of the questions’ first guesses, with rank 1 indicating the question with the lowest MSE and rank 120 indicating the question with the highest MSE. Our primary hypothesis concerned the interaction between the perspective-taking manipulation (disagreeing perspective vs. self perspective) and the difficulty of the questions, ranging from the easiest to the most difficult question. More specifically, we expected that, in general, the benefit of averaging would increase as a function of question difficulty. with more benefit for increasingly difficult questions, yet this pattern was expected to be more pronounced when aggregating first guesses with second estimates made from a disagreeing perspective (vs. simply aggregating first guesses with second guesses). Please note that this analysis was exploratory in Experiment 1a but was explicitly preregistered in Experiment 1b.

Although we present the results using the ranked operationalization of difficulty below, the Supplemental Material available online include findings for all experiments using two alternative operationalizations of difficulty—first, an operationalization of difficulty in terms of the observed (average) raw error in a question’s first estimates, and second, an operationalization of difficulty in terms of the observed variability in the first estimates. This latter approach differs from operationalizations based on average errors, because those do not differentiate between response bias and response variability. For example, consider a question for which the true answer is 55%, but nearly everyone believes it to be around 20% (indicating a high degree of bias, but a low degree of response variability). Compare this with a question for which the true answer is also 55%, but responses are spread across the entire range of the available answer options (indicating a low degree of bias, but a high degree of response variability). Using observed errors to determine difficulty, the former question would likely be labeled as difficult, whereas the latter would be considered easy. Yet observing a high degree of variability in people’s responses could also be seen as a reliable sign that the question is difficult to answer. Hence, in the Supplemental Material, we report the results of an approach that uses variability (as opposed to error) as its basis for determining a question’s level of difficulty. The results remain qualitatively the same when using these alternate operationalizations of difficulty.

### Results

For all analyses, unless stated otherwise, we employed Bayesian fully maximal random-effects mixed-effect models^3^ (following Fiechter, 2024). We first compared the degree of bracketing between the self-perspective and disagreeing-perspective conditions (i.e., how often first and second estimates land on opposite sides of a question’s true answer). The results showed less bracketing in the self-perspective condition in both experiments (Experiment 1a: estimate = −0.81, error = 0.06, 95% credible interval, or CrI = [−0.94, −0.69]; Experiment 1b: estimate = −0.88, error = 0.06, 95% CrI = [−1.01, −0.76]). Although 21% (18%) of the estimates in the self-perspective condition bracketed true answers, 36% (42%) were bracketed in the disagreeing-perspective condition in Experiment 1a (Experiment 1b). Similarly, Pearson correlations between estimates in the disagreeing-perspective and self-perspective conditions indicated that taking a disagreeing perspective produced more diverse, independent estimates (Experiment 1a: *r*_disagree_ = .33 vs. *r*_self_ = .74; Experiment 1b: *r*_disagree_ = .33 vs. *r*_self_ = .77). According to earlier studies and theoretical work, these two observations should, in principle, improve the accuracy of average estimates.

**Fig. 1.**
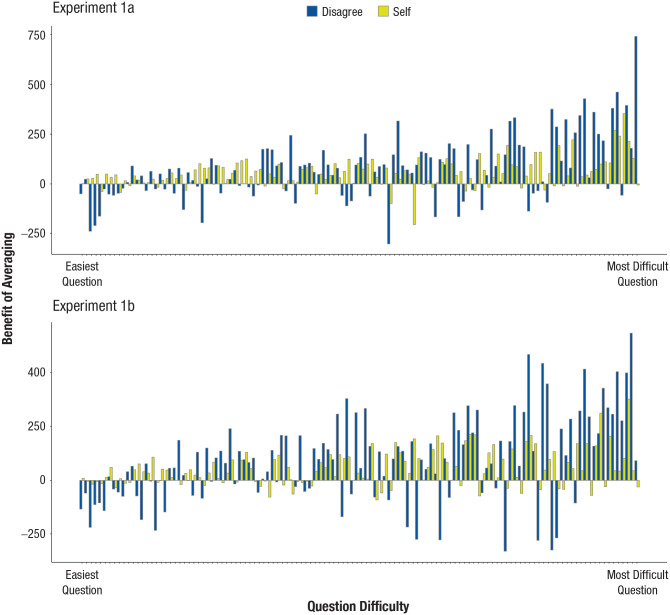
Benefit of averaging as a function of question difficulty (rank order) and perspective taking (disagree vs. self) for Experiments 1a (top) and 1b (bottom). The benefit of averaging is computed by subtracting the squared error of the average estimate from the squared error associated with a person’s first estimate—the higher the value, the more benefit there is to averaging two estimates (i.e., the more accurate the aggregate). Each bar represents the benefit of averaging for a particular question by condition. To assess question difficulty, we ranked all 120 questions from easiest to most difficult on the basis of the mean squared error of the questions’ first guesses. Note that this ranking is conducted separately for each experiment, meaning that the order of the questions in terms of difficulty is not identical across experiments (although there is a considerable overlap between experiments in which questions were deemed easier or more difficult; for more details, see the question list with the rankings for each experiment on the Open Science Framework page).

In Experiment 1a, aligned with Fiechter’s (2024) findings, there was no overall benefit of averaging when taking a disagreeing perspective (Experiment 1a: estimate = −19.31, error = 16.24, 95% CrI = [−51.90, 13.09]) with an average benefit of 76.8 in the disagreeing condition and 56.6 in the self-perspective condition. In Experiment 1b, however, there was a benefit of averaging when taking a disagreeing perspective, albeit with relatively wide credible intervals (Experiment 1b: estimate = −29.75, error = 14.90, 95% CrI = [−59.23, −0.59]) with an average benefit of 73.2 in the disagreeing condition and 44.1 in the self-perspective condition. We subsequently tested the main prediction of interest, namely the interaction between question difficulty and perspective taking. In both experiments, the interaction estimates’ credible intervals did not include zero (Experiment 1a: estimate = −1.65, error = 0.44, 95% CrI = [−2.51, −0.78]; Experiment 1b: estimate = −1.42, error = 0.43, 95% CrI = [−2.26, −0.59]). As Figure 1 shows, although the benefit of averaging increased with increasingly difficult questions, this effect was much more pronounced when taking a disagreeing perspective (Experiment 1a: estimate = 2.32, error = 0.37, 95% CrI = [1.61, 3.07]; Experiment 1b: estimate = 2.10, error = 0.39, 95% CrI = [1.31, 2.87]) as opposed to when second guesses were made from one’s own point of view (Experiment 1a: estimate = 0.68, error = 0.24, 95% CrI = [.21, 1.15]; Experiment 1b: estimate = 0.68, error = 0.22, 95% CrI = [.25, 1.11]). Note that averaging first estimates with estimates made from a disagreeing perspective was particularly beneficial for the most difficult questions (vs. adding a second guess to one’s initial estimate) and less beneficial for the easier ones (see Table 1).

**Table 1.** Percentage of questions (with the actual ratio of questions in brackets) in which the disagreeing-perspective condition outperformed the self-perspective condition (and vice versa). Results are displayed across all questions and separately across the different levels of question difficulty (binned into four levels following the rankings, from the easiest questions to the most difficult)

Questions	Experiment 1a		Experiment 1b		Experiment 2	
	Disagreeing-perspective condition	Self-perspective condition	Disagreeing-perspective condition	Self-perspective condition	Disagreeing-perspective condition	Self-perspective condition
All	52.5% (63/120)	47.5% (57/120)	55.0% (66/120)	45.0% (54/120)	60.0% (18/30)	40.0% (12/30)
Most easy	36.7% (11/30)	63.3% (19/30)	30.0% (9/30)	70.0% (21/30)	33.3% (5/15)	66.7% (10/15)
Moderately easy	40.0% (12/30)	60.0% (18/30)	70.0% (21/30)	30.0% (9/30)	/	/
Moderately difficult	66.7% (20/30)	33.3% (10/30)	46.7% (14/30)	53.3% (16/30)	/	/
Most difficult	66.7% (20/30)	33.3% (10/30)	73.3% (22/30)	26.7% (8/30)	86.7% (13/15)	13.3% (2/15)

Note: There is no information on moderately easy or difficult questions for Experiment 2 because in this experiment 30 questions were preselected from the pool of questions from the other experiments, where 15 were categorized as easy and 15 were categorized as difficult.

## Experiment 2

### Method

Using Prolific, we recruited 501 participants—all from the United Kingdom and the United States, with English as their first language. Participants were paid a fixed amount for their participation. We excluded from participation anyone who had participated in similar experiments we ran on the Prolific platform. Following our preregistration plan, we excluded those participants from the analyses who reported that they had looked up any of the answers, resulting in a final sample of 495 participants from the United Kingdom and the United States (age: *Mdn* = 39 years, IQR = 20.5 years; 58% female). Experiment 2 was identical to Experiments 1a and 1b, with two significant changes. First, as opposed to using 120 questions, the current experiment had 30 questions. Second, whereas we determined the difficulty ranking in Experiments 1a and 1b a posteriori, in the current experiment we predetermined (and preregistered) the difficulty order of the set of 30 questions before collecting the data. Fifteen of the questions were classified as easy (i.e., with a ranking from 1 to 15), and another 15 were classified as difficult (i.e., with a ranking from 16 to 30). These predetermined rankings are shown in the Excel file attached to the preregistration. We determined the rank order of these 30 questions by using the questions’ rankings obtained in Experiments 1a and 1b. Note that we observed a considerable overlap across these experiments regarding which of the 30 questions were deemed easiest and which most difficult. Because there were 30 questions, following the R(NCC) design, we devised three batches with 10 questions each. Ethical approval was obtained from the ethical review board at Eindhoven University of Technology.

### Results

Similar to Experiments 1a and 1b, the results again showed less bracketing in the self-perspective condition (estimate = −0.74, error = 0.12, 95% CrI = [−0.99, −0.49]): 22% of the estimates in the self-perspective condition bracketed true answers, compared with 35% in the disagreeing-perspective condition. Similarly, correlations between estimates in the disagreeing- and self-perspective conditions indicated that taking a disagreeing perspective produced more diverse, independent estimates (Pearson *r*_disagree_ = .33 vs. *r*_self_ = .78). Similar to Experiment 1a, there was again no overall benefit of averaging when taking a disagreeing perspective (estimate = −51.45, error = 31.93, 95% CrI = [−114.13, 12.24]), with an average benefit of 108.0 in the disagreeing-perspective condition and 57.6 in the self-perspective condition.

**Fig. 2.**
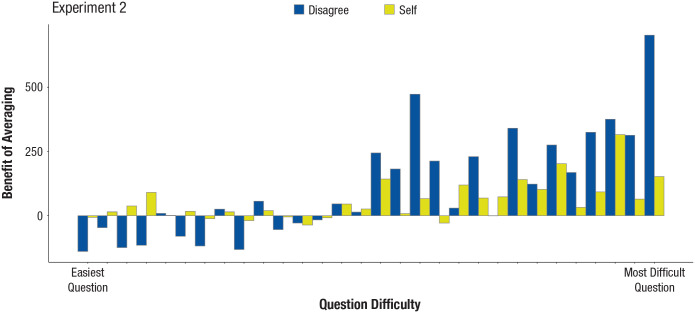
Benefit of averaging as a function of question difficulty (rank order) and perspective taking (disagreeing perspective vs. self perspective). The benefit of averaging is computed by subtracting the squared error of the average estimate from the squared error associated with a person’s first estimate—the higher the value, the more benefit there is to averaging two estimates (i.e., the more accurate the aggregate). Each bar represents the benefit of averaging for a particular question by condition. To assess question difficulty, we predetermined the difficulty order of a set of 30 questions. Rankings 1 to 15 were classified as easy questions, and rankings 16 to 30 were classified as difficult questions. Note that the substantially larger benefit observed in questions 16 to 30 is thus due to the fact that these questions are ranked as much more difficult in the predetermined order than questions 1 to 15.

Importantly, using the preregistered difficulty order, we tested the interaction between question difficulty and perspective taking. Figure 2 displays the main results. The interaction estimate’s credible interval again did not include zero (estimate = −13.49, error = 3.15, 95% CrI = [−19.77, −7.34]). There was a larger benefit of averaging when taking a disagreeing perspective (estimate = 18.75, error = 2.79, 95% CrI = [13.28, 24.27]) as opposed to a self perspective (estimate = 5.26, error = 1.97, 95% CrI = [1.42, 9.17]) as question difficulty increased (see also Table 1, Experiment 2 for a breakdown of the benefit (or not) of taking a disagreeing perspective across the different levels of question difficulty).

## General Discussion

The presented results support the notion that combining first guesses with estimates that are markedly different does not, by default, improve the accuracy of inner crowds (Gaertig & Simmons, 2021; Van de Calseyde & Efendić, 2022). Applying a maximum random-effect structure model that explicitly takes into account item variability, we observed mixed results, in general, of the benefit of averaging between the self-perspective and the disagreeing-perspective conditions. Specifically, although we did not find an overall effect between these conditions in Experiments 1a and 2, there was an effect in Experiment 1b, albeit with wide credible intervals hinting at the instability of the overall effect. These findings are consistent with the reanalysis by Fiechter (2024) of new data with a substantially larger number of questions. Furthermore, these findings again reinforce the risks of failing to account for item-level variance in experimental psychology (Barr et al., 2013; Oberauer, 2022; Yarkoni, 2022).

However, based on the current analyses, it appears that the difficulty of a question is a key factor to consider when evaluating the effectiveness of taking a disagreeing perspective for the wisdom of inner crowds. Across three preregistered studies, we have demonstrated that for the most difficult questions (i.e., where people’s first guesses are typically far removed from the question’s true answer), taking a disagreeing perspective when making a second guess is very effective, producing aggregate estimates that are substantially more accurate than simply adding a second guess from one’s own perspective. Conversely, though, for questions considered to be very easy (i.e., questions for which people’s first guesses typically are already close to the question’s true answer), taking a disagreeing perspective proved ineffective or even harmful. This pattern is consistent across all three experiments. The same is true for moderately easy and moderately difficult questions, although we observed a deviation in Experiment 1b (in that experiment, taking a disagreeing perspective produced better results for moderately easy questions, whereas making a second guess produced better results for moderately difficult questions). Interestingly, these findings held when we operationalized question difficulty in terms of response variability. The benefit of the inner crowd, and particularly of taking a disagreeing perspective, appears to extend to questions for which there is high uncertainty (i.e., variability) and not just a high degree of error. It is noteworthy to mention that the average error on first guesses was highly correlated with response variability (i.e., questions that show high average errors are also the ones that show a high degree of response variability).

These overall findings may have significant consequences for work on the wisdom of inner crowds and potentially any intervention designed to enhance diversity between estimates (e.g., dialectical bootstrapping, extending time periods between estimates, or encouraging people to take a disagreeing perspective when making a second guess; Herzog & Hertwig, 2009; Van de Calseyde & Efendić, 2022; Vul & Pashler, 2008). Specifically, the current results indicate there may be such a thing as too much estimation diversity when it comes to questions that people generally find easier. In such cases, combining multiple estimates from the same individual may be unnecessary or even harmful. It thus follows that interventions aimed at improving average estimates must be targeted to be effective (i.e., only applied to specific estimation problems) rather than general (i.e., applied to all estimation problems).

Although the current instructions required people to respond to a question twice, it is worth noting that inner-crowd benefits are even greater when people provide more than two estimates (e.g., Fiechter & Kornell, 2021; van Dolder & van den Assem, 2018). However, taking a disagreeing perspective may be limited to cases in which only two estimates can be provided, as it would presumably be difficult to adopt multiple disagreeing perspectives over three or more guesses. Likewise, please note that the presented results were obtained through the recruitment of online participants from the United States and the United Kingdom. Future work should confirm whether these findings hold true for other samples. Finally, although people were prompted to take the perspective of someone they disagree with, it is unclear whether they, following our instructions, actually went through the process of adopting such a perspective. The original article reported the results of an experiment (Experiment 3) showing that taking a disagreeing perspective helps people deanchor more readily from their first estimates by having them consider and adopt more extreme answers—answers they would never have considered if they had adhered to their own point of view (Van de Calseyde & Efendić, 2022). Yet an alternative mechanism that may have encouraged this process of deanchoring is that people simply provided a radically different estimate without actually engaging in perspective taking. Future research should aim to disentangle, in more detail, whether prompting people to take a disagreeing perspective indeed leads them to adopt such a perspective. One possible approach could be to have participants go through a step-by-step procedure (similar to dialectical bootstrapping; Herzog & Hertwig, 2014) in which they explicitly name the disagreeing individual and clearly list this individual’s thought process before answering the estimation question.

On a final note: In the current analyses, we assessed whether a question was relatively easy or difficult by observing how far, on average, people’s first guesses were removed from the question’s true answer, as well as how variable people’s first guesses were (i.e., difficulty in terms of response variability; see the Supplemental Material). For determining a question’s level of difficulty such approaches are typically impractical because they require (a) knowing how others responded to the question, (b) knowing the true answer (when operationalizing difficulty in terms of average error), and (c) receiving a large number of responses. These features are usually unavailable to individuals. Future research should explore the possibility of identifying which questions are easy or difficult for a particular individual by, for example, relying on cues that have been shown to correlate with accuracy (e.g., a person’s degree of confidence in one’s answer or the time needed to answer a question; Kämmer et al., 2017; Koriat, 2012; Sherbino et al., 2012; Staal et al., 2021). This would help devise prompts that could assist individuals in determining whether to follow their first guess, to make a second guess, or to complement their first guess with a guess taken from a disagreeing perspective.

## Supplemental Material

sj-docx-1-pss-10.1177_09567976251325518 – Supplemental material for Disagreeing Perspectives Enhance Inner-Crowd Wisdom for Difficult (but Not Easy) Questions Supplemental material, sj-docx-1-pss-10.1177_09567976251325518 for Disagreeing Perspectives Enhance Inner-Crowd Wisdom for Difficult (but Not Easy) Questions by Philippe P. F. M. Van de Calseyde and Emir Efendić in Psychological Science

## Transparency

*Action Editor:* Gabriele Paolacci

*Editor:* Simine Vazire

*Author Contributions*

**Philippe P. F. M. Van de Calseyde:** Conceptualization, Data curation, Formal analysis, Investigation, Methodology, Project administration, Writing – original draft, Writing – review & editing.

**Emir Efendić:** Conceptualization, Data curation, Formal analysis, Investigation, Methodology, Project administration, Writing – original draft, Writing – review & editing.

**Artificial Intelligence:** ChatGPT was used on some sections of the text to improve grammar and to rephrase certain sections. No other artificial-intelligence-assisted technologies were used in this research or the creation of this article.

**Ethics:** Ethical approval was obtained from the ethical review board at Eindhoven University of Technology (Reference No. ERB2020IEIS29).

**Open Practices:** Experiment 1a disclosures. Preregistration: The hypotheses and methods were preregistered, and the analysis plan was partly preregistered (https://doi.org/10.17605/OSF.IO/A8DEH) on December 7, 2023, prior to data collection, which began on December 7, 2023. The analyses discerning the relative ease versus difficulty of questions were not preregistered. There were two minor deviations from the preregistered plan. First, the preregistered models for the interaction test included a random effect of item difficulty for items, but item difficulty varies for participants, not items. The models in the manuscript correctly specify the random structure. Second, the preregistration specified a frequentist mixed-effects model with lmer, whereas a Bayesian mixed model with brms was used in the manuscript. Note that the preregistered power analysis was based on a planned recruited sample, but the analyzed sample was different because of exclusions. Materials: All study materials are publicly available (https://doi.org/10.17605/OSF.IO/SZRHD). Data: All primary data are publicly available (https://doi.org/10.17605/OSF.IO/68E4P). Analysis scripts: All analysis scripts are publicly available (https://doi.org/10.17605/OSF.IO/GJBKQ). Computational reproducibility: The computational reproducibility of the results has been independently confirmed by the journal’s STAR team. Experiment 1b disclosures. Preregistration: The hypotheses and methods and analysis plan were preregistered (https://doi.org/10.17605/OSF.IO/4AGXJ) on April 2, 2024, prior to data collection, which began on April 4, 2024. There was one minor deviation from the preregistered plan. The preregistered models for the interaction test included a random effect of item difficulty for items, but item difficulty varies for participants, not items. The models in the manuscript correctly specify the random structure. Note that the preregistered power analysis was based on a planned recruited sample, but the analyzed sample was different because of exclusions. Materials: All study materials are publicly available (https://doi.org/10.17605/OSF.IO/SZRHD). Data: All primary data are publicly available (https://doi.org/10.17605/OSF.IO/68E4P). Analysis scripts: All analysis scripts are publicly available (https://doi.org/10.17605/OSF.IO/GJBKQ). Computational reproducibility: The computational reproducibility of the results has been independently confirmed by the journal’s STAR team. Experiment 2 disclosures. Preregistration: The hypotheses and methods and analysis plan were preregistered (https://doi.org/10.17605/OSF.IO/C7VJF) on April 11, 2024, prior to data collection, which began on April 12, 2024. There was one minor deviation from the preregistered plan. The preregistered models for the interaction test included a random effect of item difficulty for items, but item difficulty varies for participants, not items. The models in the manuscript correctly specify the random structure. Materials: All study materials are publicly available (https://doi.org/10.17605/OSF.IO/SZRHD). Data: All primary data are publicly available (https://doi.org/10.17605/OSF.IO/68E4P). Analysis scripts: All analysis scripts are publicly available (https://doi.org/10.17605/OSF.IO/GJBKQ). Computational reproducibility: The computational reproducibility of the results has been independently confirmed by the journal’s STAR team.

## Notes

1. Participants were asked: “Did you look up any of the answers to the question?” and told to be honest in answering, as they would receive participation payment regardless of their response.

2. We relied on the accompanying Shiny app from Judd et al. (2017) to obtain these calculations: https://jakewestfall.shinyapps.io/two_factor_power/

3. The preregistered models for the interaction test included a random effect of item difficulty for items—but item difficulty varies for participants, not items. The models in the manuscript correctly specify the random structure. Both specifications have qualitatively the same results.
