# Supplementary material for: Disagreeing Perspectives Enhance Inner-Crowd Wisdom for Difficult (but Not Easy) Questions
Source: Psychol Sci. 2025 Mar 24;36(3):147–56. doi: 10.1177/09567976251325518 (PMC13428925; doi:10.1177/09567976251325518)
Supplement: sj-docx-1-pss-10.1177_09567976251325518 – Supplemental material for Disagreeing Perspectives Enhance Inner-Crowd Wisdom for Difficult (but Not Easy) Questions [file sj-docx-1-pss-10.1177_09567976251325518.docx]

**Supplementary Materials**

*for*

Disagreeing perspectives enhance inner crowd wisdom for difficult (but not easy) questions

**Operationalization of difficulty as the observed raw error of first estimates**

In this section, we tested the interaction between question difficulty and perspective taking with question difficulty being defined here as the observed MSEs on the questions’ first estimates. Specifically, unlike in the main text, we take the raw values as our predictor in the model instead of creating and using their ranking. For Experiment 2, we additionally ran what we call an “out-of-sample” analysis. Specifically, instead of using observed raw errors from Experiment 2, we used the observed raw errors from our prior Experiments 1A and 1B (i.e., average first-guess error of both experiments) to define question difficulty a-priori. In all three experiments, the interaction estimates’ credible intervals did not include zero:

- Experiment 1A: estimate = -1.65, error = 0.44, 95% CrI [-2.51; -0.78];
- Experiment 1B: estimate = -1.42, error = 0.43, 95% CrI [-2.26; -0.59]);
- Experiment 2: estimate = -0.22, error = 0.05, 95% CrI [-.32; -.12]);
- Experiment 2 (out-of-sample): estimate = -0.21, error = 0.05, 95% CrI [-.31; -.12]);

As Figure S1 shows, while the benefit of averaging increased with increasingly more difficult questions, this effect was much more pronounced when taking a disagreeing perspective:

- Experiment 1A: estimate = 0.28, error = 0.04, 95% CrI [.20; .36];
- Experiment 1B: estimate = 0.25, error = 0.04, 95% CrI [.16; .32]);
- Experiment 2: estimate = 0.31, error = 0.04, 95% CrI [.23; .39]);
- Experiment 2 (out-of-sample): estimate = 0.30, error = 0.04, 95% CrI [.22; .38]);

as opposed to when second guesses were made from one’s own point of view.

- Experiment 1A: estimate = 0.08, error = 0.03, 95% CrI [.03; 0.14];
- Experiment 1B: estimate = 0.07, error = 0.03, 95% CrI [.02; .12]);
- Experiment 2: estimate = 0.09, error = 0.03, 95% CrI [.03; .15]);
- Experiment 2 (out-of-sample): estimate = 0.09, error = 0.03, 95% CrI [.03; .15]);

**
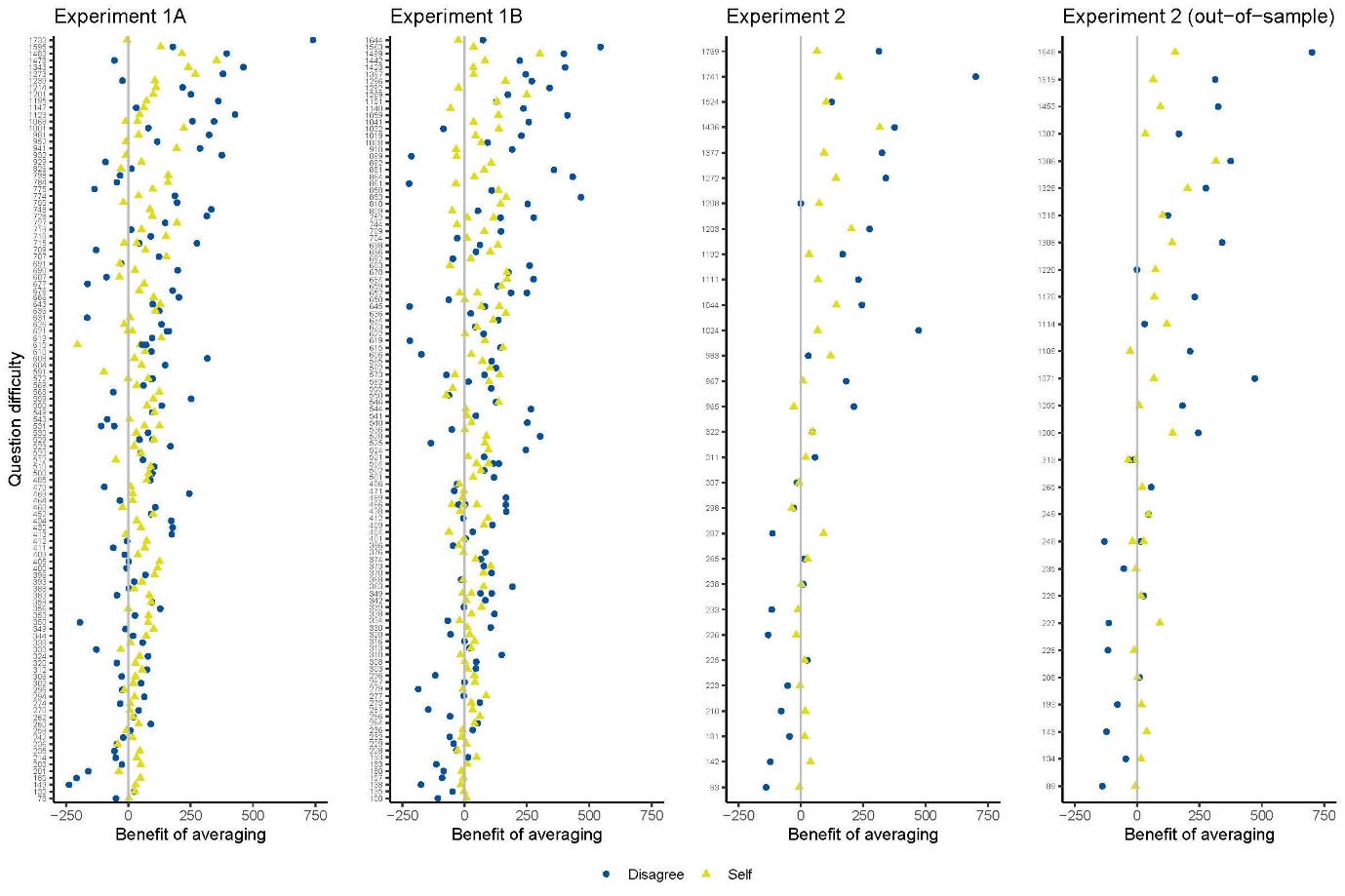
**

**Fig. S1.** Benefit of averaging as a function of question difficulty (observed MSEs on questions’ first estimates) and perspective taking (Disagree vs. Self) for Experiments 1A, 1B, and 2. For Experiment 2, (out-of-sample) question difficulty is defined a-prior using the observed raw errors from our prior Experiments 1A and 1B. Benefit of averaging is computed by subtracting the squared error of the average estimate from the squared error associated with a person’s first estimate – the higher the value, the more benefit there is to averaging two estimates i.e., the more accurate the aggregate (the 0 line would indicate no benefit). Each point represents the benefit of averaging for a particular question by condition. Note: Experiment 2 had only 30 questions while Experiments 1A and 1B included 120 questions.

**Operationalization of difficulty as the observed variability of first estimates**

In this section, we tested the interaction between question difficulty and perspective taking. However, question difficulty here is defined as the observed response variability of first estimates. Specifically, unlike in the main text, to obtain a difficulty ranking we use the variability in people’s first estimates on a question (as expressed by their variance), with more variability across estimates being a proxy for a more difficult question. Indeed, the correlation between response variability on first estimates and their corresponding MSE was quite high across all three Experiments (Ex1A: .61; Ex1B: .58; Ex2: .75, all *p* values < .001). For Experiment 2, we additionally ran what we call an “out-of-sample” analysis. Specifically, instead of using observed response variability from Experiment 2, we used the observed response variability from our prior Experiments 1A and 1B (i.e., average first-guess variability of both experiments) to define question difficulty a-priori. As with all the other operationalizations of difficulty, in all three experiments, the interaction estimates’ credible intervals did not include zero:

- Experiment 1A: estimate = -1.43, error = 0.43, 95% CrI [-2.28; -0.58]);
- Experiment 1B: estimate = -1.55, error = 0.41, 95% CrI [-2.35; -0.75]);
- Experiment 2: estimate = -14.56, error = 2.72, 95% CrI [-19.96; -9.27]);
- Experiment 2 (out-of-sample): estimate = -12.53, error = 3.01, 95% CrI [-18.47; -6.67]);

As Figure S2 shows, while the benefit of averaging increased with increasingly more difficult questions, this effect was much more pronounced when taking a disagreeing perspective:

- Experiment 1A: estimate = 2.18, error = 0.37, 95% CrI [1.45; 2.90];
- Experiment 1B: estimate = 2.29, error = 0.38, 95% CrI [1.55; 3.02]);
- Experiment 2: estimate = 19.45, error = 2.44, 95% CrI [14.67; 24.32]);
- Experiment 2 (out-of-sample): estimate = 16.60, error = 3.15, 95% CrI [10.44; 22.80]);

as opposed to when second guesses were made from one’s own point of view.

- Experiment 1A: estimate = 0.75, error = 0.23, 95% CrI [.31; 1.20];
- Experiment 1B: estimate = 0.73, error = 0.21, 95% CrI [.33; 1.15]);
- Experiment 2: estimate = 4.89, error = 1.90, 95% CrI [1.17; 8.63]);
- Experiment 2 (out-of-sample): estimate = 4.07, error = 1.89, 95% CrI [0.34; 7.80]);


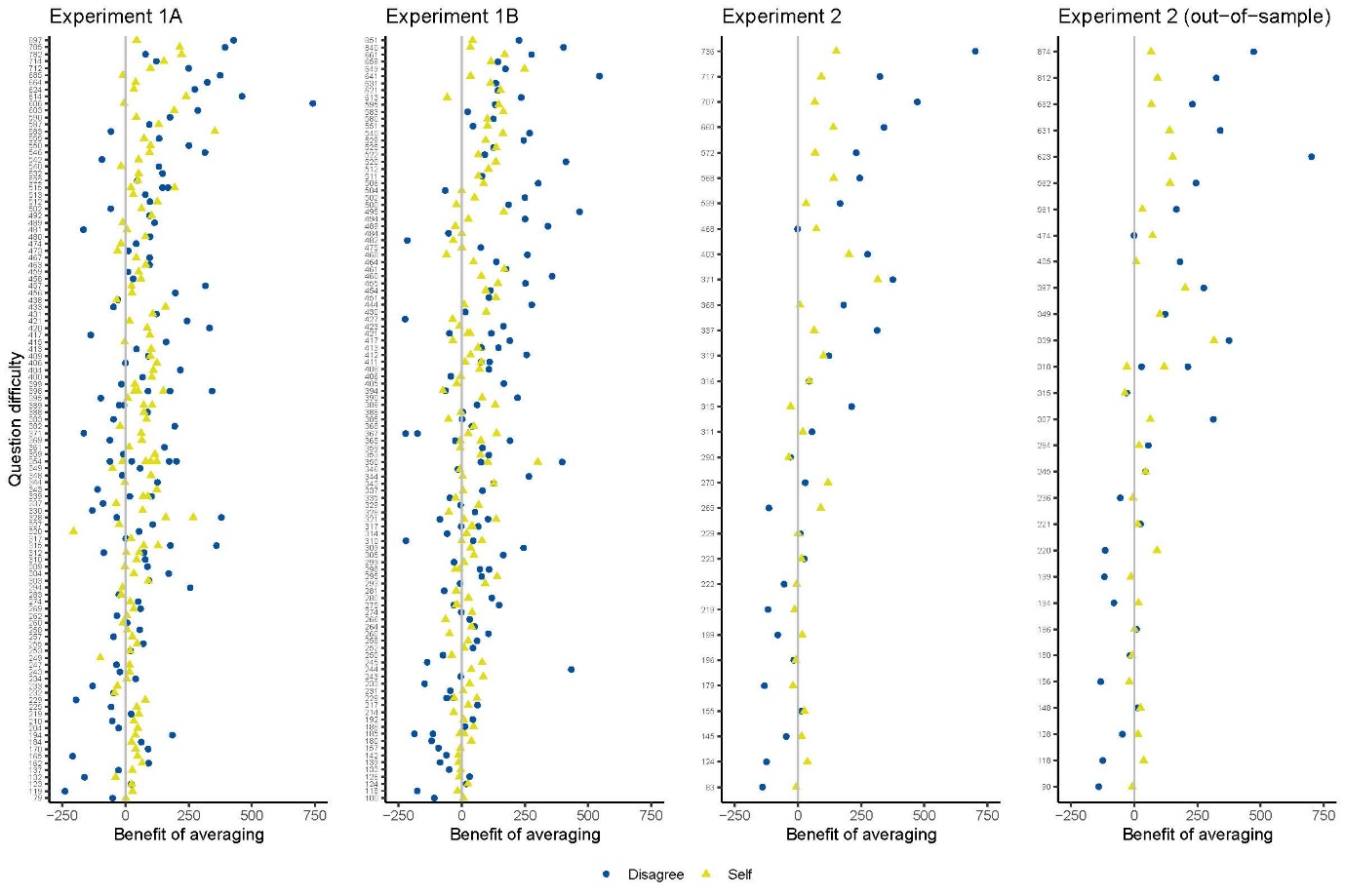


**Fig. S2**. Benefit of averaging as a function of question difficulty (observed variance across questions’ first estimates) and perspective taking (Disagree vs. Self) for Experiments 1A, 1B, and 2. For Experiment 2, (out-of-sample) question difficulty is defined a-prior using the observed response variability from our prior Experiments 1A and 1B. Benefit of averaging is computed by subtracting the squared error of the average estimate from the squared error associated with a person’s first estimate – the higher the value, the more benefit there is to averaging two estimates i.e., the more accurate the aggregate (the 0 line would indicate no benefit). Each point represents the benefit of averaging for a particular question by condition. Note: Experiment 2 had only 30 questions while Experiments 1A and 1B included 120 questions.
